# Clathrin Is Important for Virulence Factors Delivery in the Necrotrophic Fungus *Botrytis cinerea*

**DOI:** 10.3389/fpls.2021.668937

**Published:** 2021-06-16

**Authors:** Eytham Souibgui, Christophe Bruel, Mathias Choquer, Amélie de Vallée, Cindy Dieryckx, Jean William Dupuy, Marie-Pascale Latorse, Christine Rascle, Nathalie Poussereau

**Affiliations:** ^1^UMR 5240, CNRS MAP, INSA Lyon, Bayer SAS, UCBL, University Lyon, Lyon, France; ^2^Plateforme Protéome, Centre de Génomique Fonctionnelle, Université de Bordeaux, Bordeaux, France; ^3^Centre de Recherche La Dargoire Bayer SAS, Lyon, France

**Keywords:** *Botrytis cinerea*, clathrin, virulence, infection cushion, secretomics

## Abstract

Fungi are the most prevalent plant pathogens, causing annually important damages. To infect and colonize their hosts, they secrete effectors including hydrolytic enzymes able to kill and macerate plant tissues. These secreted proteins are transported from the Endoplasmic Reticulum and the Golgi apparatus to the extracellular space through intracellular vesicles. In pathogenic fungi, intracellular vesicles were described but their biogenesis and their role in virulence remain unclear. In this study, we report the essential role of clathrin heavy chain (CHC) in the pathogenicity of *Botrytis cinerea*, the agent of gray mold disease. To investigate the importance of this protein involved in coat vesicles formation in eukaryotic cells, a T-DNA insertional mutant reduced in the expression of the CHC-encoding gene, and a mutant expressing a dominant-negative form of CHC were studied. Both mutants were strongly affected in pathogenicity. Characterization of the mutants revealed altered infection cushions and an important defect in protein secretion. This study demonstrates the essential role of clathrin in the infectious process of a plant pathogenic fungus and more particularly its role in virulence factors delivery.

## Introduction

Filamentous fungi constitute the major group of crop pathogenic microbes that reduce the yield and quality of agricultural production. Currently, the fight against fungal infections is mainly based on the use of resistant cultivars and phytosanitary treatments (Fisher et al., 2018). However, new environmentally friendly crop protection methods are needed to limit the presence of residues in soil and the continual emergence of new races of fungi able to overcome chemical treatments (Shang et al., 2019). Within this context, a better understanding of the infectious process developed by plant pathogenic fungi becomes a prerequisite to develop new and alternative solutions.

Several approaches have been explored to identify the molecular actors and mechanisms involved in the infectious process of fungi. Among these, random mutagenesis leading to the production and selection of non-pathogenic mutants has been used in numerous fungal models (Betts et al., 2007; Blaise et al., 2007; Münch et al., 2011), including the gray mold fungus *Botrytis cinerea*, a major and typical necrotroph causing serious pre- and post-harvest losses in many crops (Dean et al., 2012). Using *Agrobacterium tumefaciens* mediated mutagenesis, collections of mutants have been generated, leading to the identification of genes important for the pathogenicity of *B. cinerea.* The first collection allowed the identification of new virulence-related proteins (Giesbert et al., 2012, 2014; Schumacher et al., 2019) and the second revealed the importance of the kinesin KPL7 (Tayal et al., 2017), the phosphoenolpyruvate carboxykinase PCK1 (Liu et al., 2018) and a virulence-associated protein HBF1 (Liu et al., 2019) in the infection process of the fungus. More recently, our group presented a collection of 2,144 *Agrobacterium tumefaciens*-mediated transformation (ATMT) mutants (de Vallée et al., 2019). The characterization of twelve non-pathogenic mutants in this collection revealed common traits such as a disturbance of the secretome and an altered differentiation of infection cushions (penetration structures of the pathogen). Another mutant in this collection held our attention. This strain was altered in pathogenicity and mutated in the promoter of the clathrin heavy chain-encoding gene, a gene whose role in the infection process of phytopathogenic fungi has never been described.

Discovered in mammalian cells (Pearse, 1976), clathrin is a protein complex conserved across all eukaryotic organisms and is mainly involved in the formation of coated vesicles at both the plasma membrane (PM) and the *trans*-Golgi network (TGN). Several reports describe the isolation and role of clathrin coat vesicles (CCV) in mammalian, plant, protozoan parasites and yeast cells (see for review, Coleman et al., 1988; Karsten et al., 1998; Bannister et al., 2000; Pieperhoff et al., 2013; Robinson, 2015). Clathrin has been the subject of numerous studies and its roles in cellular processes are still under investigations (for review Robinson, 2015; Ekanayake et al., 2019; Briant et al., 2020). It requires the assembly of three clathrin heavy chains (CHC) associated with three clathrin light chains (CLC) to form triskelions that are recruited to appropriate membranes through adaptor complexes linked to cargoes. The assembled clathrin lattice results in CCV that detach from the membrane and carry the cargoes to their destinations. Accessory proteins participate in vesicle scission and their subsequent uncoating in the cytoplasm (Robinson, 2015). In many unicellular and multicellular eukaryotes, the role of CCV in clathrin-mediated endocytosis (CME) is well established (McMahon and Boucrot, 2011); they are involved in the intake of extracellular and membrane compounds. In connection with this, they are also exploited by bacterial pathogens and viruses to gain access into cells (Robinson et al., 2018; Latomanski and Newton, 2019; Yang and Shen, 2020). At the TGN, CCV are implicated in the secretion of newly synthesized proteins [Gurunathan et al., 2002 (yeast); Borgonovo et al., 2006 (human); Burgess et al., 2011 (drosophila)] and in protein trafficking to the vacuole, *via* the endosome (Stahlschmidt et al., 2014).

Clathrin mutants have been generated in several pathogenic microorganisms to investigate the importance of this protein in virulence. In *Trypanosoma brucei*, depletion of clathrin by antisense RNA resulted in rapid lethality at the bloodstream stage of the parasite. The flagellar pocket, the site of both endocytosis and exocytosis in this protist, became massively enlarged, suggesting a defect in endocytosis (Allen et al., 2003). In the case of the parasite *Toxoplasma gondii*, Pieperhoff et al. (2013) showed that dominant negative mutants, termed HUB mutants, were not affected in host cell invasion but exhibited an aberrant development inside the host cell. More recently, the encapsulated yeast *Cryptococcus neoformans* lacking the CHC-encoding gene was shown defective in the uptake and trafficking of hemoglobin, the major source of iron for this fungal pathogen, and defective in the production of two key virulence factors, namely capsule and melanin (Bairwa et al., 2019).

To date, little information is available about clathrin in filamentous fungi. A single report describes the purification and visualization of CCV in the saprophytic fungus *Neurospora crassa* (Rosa and Maccioni, 1987). As to the role of clathrin, it was only studied in *Aspergillus nidulans* (Schultzhaus et al., 2017) and more recently in *Sclerotinia sclerotiorum* (Wytinck et al., 2020). In the saprophytic fungus *A. nidulans*, CHC was localized to the PM, the TNG and to small puncta trafficking long distance in hyphae. The CHC-encoding gene was identified as essential and the deletion of the CLC-encoding gene led to a strongly altered fungal growth and development (Schultzhaus et al., 2017). In the phytopathogenic fungus *S. sclerotiorum*, chemicals inhibitors and RNAi-mediated knockdown mutants allowed the demonstration of clathrin mediating endocytosis of exogenous double-stranded RNA (Wytinck et al., 2020), but did not inform on the role clathrin might play in fungal pathogenicity. To date, the role of clathrin remains evasive in filamentous fungi.

Since CHC is essential in many organisms (Royle, 2006), over-production of the C-terminal third domain of CHC, termed the HUB domain, was developed in mammalian cells (Liu et al., 1998), plant cells (Adam et al., 2012) and protists (Pieperhoff et al., 2013) to alter clathrin function. Through its competition with the endogenous CHC for the binding of CLC, the over-produced HUB fragment disturbs the formation of triskelions, therefore that of clathrin coats, and hence the associated cellular functions. In this study, we used both a mutant exhibiting an insertion of the T-DNA in the promoter of the CHC gene, leading to reduced gene expression, and a HUB dominant negative genetic strategy to explore the role of CHC in the filamentous fungus *B. cinerea*. The characterization of the mutants revealed an important defect in the secretion of effectors involved in the infectious process of the pathogen.

## Materials and Methods

### Strains and Culture Conditions

*Botrytis cinerea* strain B05.10 was used as the reference strain and was used for genetic modifications. Strain T2.16 was isolated from a mutant library generated by random insertional mutagenesis using *Agrobacterium tumefaciens*-mediated transformation (de Vallée et al., 2019). The dominant negative HUB mutants were constructed using the C-terminal domain of CHC. The corresponding DNA fragment (2,198 pb) was amplified from genomic DNA of B05-10 using primers P6 and P8 (Supplementary Table 1) and fused with the *oliC* promoter of *A. nidulans* (p*oliC*) by double joint PCR (Yu et al., 2004). The p*oliC*:HUB fragment was cloned into the *Agrobacterium tumefaciens* transformation vector pBHT2 (Mullins et al., 2001) to form the pBHT2:HUB plasmid. The construction was verified by DNA sequencing and cloned into *Agrobacterium tumefaciens* to transform B05-10 using the protocol described by Rolland et al. (2003). All the strains were maintained on solid sporulation medium as previously described (Antal et al., 2012), supplemented when necessary with hygromycin B (70 μg/ml). Mycelial plugs (4-mm diameter) or conidia served as inocula and the cultures were incubated in the dark at 21°C. Transformants were purified through several rounds of single spore isolation. The absence of parental nuclei was verified by PCR using primers pair P1/P2 (Supplementary Table 1).

### Southern Blot, Rescue-PCR, Gene Expression Analysis

For DNA preparations, mycelia were grown for 3 days on solid sporulation medium overlaid with cellophane membranes. Genomic DNA was isolated using the DNeasy plant mini kit (Qiagen). DNA flanking regions were identified using rescue-PCR (de Vallée et al., 2019). Southern blot analysis was performed to precise the number of T-DNA insertion sites, as described by Rascle et al. (2018). The restriction enzyme *Nco*I was used to digest the DNA. The PCR DIG Probe Synthesis Kit (Roche) was used to label the *hph* gene [amplified with primers hph15/hph21 (Supplementary Table 1)].

For gene expression analysis by qPCR, 50 μl-droplets of conidia suspensions (10^5^spores/ml) were deposited on Teflon membranes. After incubation in a humid chamber at 21°C for 6, 16, and 24 h, RNA was extracted from 4 mg of lyophilized grounded material using the RNeasy Midi Kit (Qiagen). RT-qPCR experiments were performed as described by Rascle et al. (2018) using ABI-7900 Applied Biosystems (Applied Biosystems). Amplification reactions were carried out using SYBR Green PCR Master Mix (Applied Biosystems). Three independent biological replicates were performed. Relative quantification was based on the 2^–ΔΔC(T)^ method (Livak and Schmittgen, 2001) using pyruvate dehydrogenase (*Bcpda1*, Bcin07g01890), actin (*BcactA*, Bcin16g02020) and elongation factor (*Bcef1a*, Bcin09g05760) encoding genes as normalization internal controls. At least three independent biological replicates were analyzed. Primers P3 and P4 used for qPCR are listed in Supplementary Table 1.

For Northern analysis, conidia suspensions (10^5^ spores/ml) were spread over cellophane sheets overlaying solid sporulation medium and cells were grown for 3 days. RNA were extracted using the RNeasy Midi Kit (Qiagen) and 15 μg were fractionated by agarose-formaldehyde gel electrophoresis (under denaturing conditions) and then transferred onto a Nytran membrane using TurboBlotter device (GE Healthcare). Biotin-dsRNA probes were synthetized and labeled as follows: briefly, T7 promoter sequences were added to both ends of DNA fragments by PCR using primers pairs HUB-F/HUB-R and 28S-F/28S-R (Supplementary Table 1) to generate HUB and 28S DNA templates, respectively. The transcription reaction was then achieved using the Megascript RNAi Kit (Ambion), according to manufacturer’s instructions with some modifications: 2 μl Biotin RNA labeling mix 10× (Roche) were added to the reaction mixture instead of the dNTP solutions. After overnight incubation at 37°C, the labeled dsRNAs were digested with DNaseI and RNase and purified following manufacturer’s instructions. Hybridization was carried out in Perfect HybPlus Hybridization Buffer (Sigma) at 65°C overnight using 100 ng of biotin-labeled probe previously denatured 5 min at 95°C in presence of 10 mM EDTA. The Chemiluminescent Nucleic Acid Detection Module (Thermo Scientific) was used to detect the biotin-labeled fragments following manufacturer’s instructions and using Chemidoc XRS + camera (Bio-Rad).

### Pathogenicity Assays and Phenotypic Analyses

Infection assays were performed using primary French bean leaves (*Phaseolus vulgaris* var Saxa), wounded apple fruit (var “Goldrush”) and 1-week-old cucumber cotyledons (*Cucumis sativus*). The plants were inoculated with 4-mm agar plugs collected from 3-day-old cultures (solid sporulation medium) or conidia suspensions (1,500 spores in 7.5 μl of Gamborg B5 medium (Duchefa) supplemented with 2% glucose). Infected plants were incubated at 21°C under 80% relative humidity and dark-light (16 h/8 h) conditions. Symptoms were scored up to 7 days post inoculation (dpi) and at least three independent biological replicates were assessed.

Radial growth was measured daily following inoculation of solid sporulation medium with 4-mm mycelial plugs and incubation at 21°C in the dark. Medium acidification was tested by growing the strains on solid complete medium (Schumacher et al., 2014) adjusted to pH 8.0 and supplemented with bromothymol blue 0.01%. For each test, three independent biological experiments and statistical analyses (Student’s *t*-test) were performed. Infection cushions were observed by inverted microscopy 3 days after inoculation of agar plugs (2-mm) in 50 μl PDB 1/4 (Potato Dextrose Broth diluted to the fourth) in 24-well plates (Thermo Scientific).

### Activities of Secreted Enzymes

100 ml of CCPX medium (400 mg/L Gamborg B5 medium (Duchefa), 0.1% carboxy-methyl-cellulose (Sigma), 0.1% casein (Sigma), 0.1% polygalacturonic acid (Sigma), 0.1% xylan (Roth), pH 5.5) were inoculated with 10^7^ conidia from 11-day-old cultures (solid sporulation medium) and shaken (110 rpm) at 21°C in the dark for 72 h. Mycelia were recovered by centrifugation (1 h, 20,000 *g*, 4°C), lyophilized and weighed. Culture supernatants were frozen in liquid nitrogen and kept at −20°C before being used as samples in the following enzymatic reactions and proteomic analysis. Enzymatic activities were performed as described by de Vallée et al. (2019). Briefly, xylanase and cellulase activities were recorded on a plate reader TECAN infinite M1000 using the EnzChek-Ultra-xylanase assay kit (Thermofisher) and the cellulase assay kit (Abcam), respectively, according to the manufacturer’s instructions. Polygalacturonase activity was determined by incubating samples and polygalacturonic acid (100 μl, 5 mg/ml) in acetate buffer 60 mM, pH 4.0. Reactions were stopped by addition of 1 ml TBC (0.1 M Na-tetraborate, 0.1 M boric acid, 0.1% cyanoacetamide) and heating (100°C) for 10 min. Free galacturonic acid was detected by spectrophotometry at 270 nm (Molecular Devices Spectramax-485) and quantitated from a galacturonic acid standard curve. Protease activity was measured by incubating samples with 1% hemoglobin (Sigma), pH 3.5, and reactions were stopped with 25% trichloroacetic acid. After centrifugation, supernatants were mixed with NaOH 0.5 M and absorbance at 280 nm was recorded. Laccase activity was measured by incubating the samples and the ABTS substrate (Sigma) in 230 μl of 50 mM Na-acetate buffer, pH 4.0. Oxidation of ABTS was recorded at 405 nm (Molecular Devices Spectramax-485) during 30 min at 30°C. One unit was defined as the amount of enzyme producing an increase of 0.01 OD unit per min. All activities were measured on three independent biological replicates.

### Proteomic Analysis

Three independent biological replicates of exo-proteomes from the studied strains were prepared as described for enzymatic analysis. Culture supernatants were concentrated using lyophilization, mixed with trichloroacetic acid (10% final) and incubated overnight at 4°C. The precipitated proteins were collected by centrifugation (14,000 *g*, 4°C, 20 min), washed three times with glacial acetone, suspended in Tris–HCl buffer and quantified using Qubit analysis (Thermofisher). Laemmli buffer was added to 10 μg of sample proteins before separation by SDS-PAGE 10%. After a short migration, proteins were visualized by staining with InstantBlue (Expedeon) and scanning with a ChemiDoc XRS camera (Bio-Rad). The proteins were quantified with Image Lab Software v5.0 (Bio-Rad) before shotgun analysis. Each SDS-PAGE band was cut into 1 mm × 1 mm gel pieces and proteins were reduced, alkylated and digested by trypsin as previously described (Rascle et al., 2018). Online nanoLC-MS/MS analyses were performed using an Ultimate 3,000 RSLC Nano-UPHLC system (Thermo Scientific) coupled to a nanospray Q Exactive hybrid quadrupole-Orbitrap mass spectrometer (Thermo Scientific). 500 ng of each peptide extract was loaded on a 300 μm ID × 5 mm PepMap C18 precolumn (Thermo Scientific) at a flow rate of 10 μl/min. After a 3 min desalting step, peptides were separated on a 75 μm ID × 25 cm C18 Acclaim PepMap RSLC column (Thermo Scientific) with a 4–40% linear gradient of solvent B (0.1% formic acid in 80% ACN) in 108 min. The separation flow rate was set at 300 nl/min. The mass spectrometer operated in positive ion mode at a 1.8 kV needle voltage. Data was acquired using Xcalibur 3.1 software in a data-dependent mode. MS scans (m/z 300–1,600) were recorded at a resolution of *R* = 70,000 (@ m/z 200) and an AGC target of 3 × 10^6^ ions collected within 100 ms. Dynamic exclusion was set to 30 s and top 12 ions were selected from fragmentation in HCD mode. MS/MS scans with a target value of 1 × 10^5^ ions were collected with a maximum fill time of 100 ms and a resolution of *R* = 17,500. Additionally, only + 2 and + 3 charged ions were selected for fragmentation. Other settings were as follows: no sheath and no auxiliary gas flow, heated capillary temperature, 200°C; normalized HCD collision energy of 27 eV and an isolation width of 2 m/z. Protein identification and Label-Free Quantification (LFQ) were done in Proteome Discoverer 2.4. MS Amanda 2.0, Sequest HT and Mascot 2.4 algorithms were used for protein identification in batch mode by searching against Ensembl *B. cinerea* database (12 060 entries, release 31). Two missed enzyme cleavages were allowed for the trypsin. Mass tolerances in MS and MS/MS were set to 10 ppm and 0.6 Da. Oxidation (M), acetylation (K) and deamidation (N, Q) were searched as dynamic modifications and carbamidomethylation (C) as static modification. Peptide validation was performed using Percolator algorithm (Käll et al., 2007) and only “high confidence” peptides were retained corresponding to a 1% false discovery rate at peptide level. Minora feature detector node (LFQ) was used along with the feature mapper and precursor ions quantifier. The normalization parameters were selected as follows: (1) Unique peptides, (2) Precursor abundance based on intensity, (3) Normalization mode: total peptide amount, (4) Protein abundance calculation: summed abundances, (5) Protein ratio calculation: pairwise ratio based. Quantitative data were considered for master proteins, quantified by a minimum of two unique peptides in the three biological replicates, with a fold changes above two in all three replicates. The mass spectrometry proteomics data have been deposited to the ProteomeXchange Consortium *via* the PRIDE (Deutsch et al., 2019) partner repository with the dataset identifier PXD023460.

### Detection of Reactive Oxygen Species

Oxidative stress was visualized during plant infection using 3,3′-diaminobenzidine (DAB) staining as described previously (Schumacher et al., 2014). Briefly, French bean leaves were infected with a drop of spore suspension (1,500 spores in 7.5 μl of Gamborg B5 medium (Duchefa) supplemented with 2% glucose), collected at 72 h post inoculation, incubated in DAB solution (0.5 mg/ml DAB in 100 mM of citric acid buffer, pH 3.7) for 2 h in darkness at room temperature and destained using boiling ethanol during 5 min. Reactive oxygen species induced a DAB oxidation and led to apparition of a brown coloration. Hyphae were then stained 4 min with a drop of 0.5% cotton blue in lactic acid, washed in distilled water and observed under a stereomicroscope (Zeiss Discovery V20).

To detect exogenous ROS accumulation secreted by fungi, mycelia were grown on cellophane overlaying solid complete medium (CM) buffered to pH 5 for 3 days. The DAB test was performed on equal quantities (25 mg) of freshly collected mycelia of the different strains according to Schumacher et al. (2014).

### Endocytosis Assay

Conidia of the T2.16, HUB.1 and parental strains were used to inoculate 2 ml of PDB 1/4 medium in microtubes (3.10^5^ spores/ml). Following 16 h of incubation at 21°C, the young mycelia were collected by centrifugation (3,500 *g*, 5 min), suspended in 500 μl of PDB 1/4 and kept on ice. The samples (20 μl) were deposited onto glass slides and immediately mixed with 20 μl of 10 μM FM4-64 FX (Sigma) in PDB 1/4. Time-course observations were performed using a confocal microscope (Zeiss LSM510: Exc 488 nm/Em 700 nm). Quantification of the endocytic process was performed using the Fiji-ImageJ software. For all studied strains, the 20 μm apical regions of 10 different hyphae were analyzed. Images were zoomed to 300%. The polygon selection tool was used to outline the plasma membranes and to measure their mean fluorescence. In parallel, the mean fluorescence of the intracellular regions was similarly measured. This operation was repeated for each time point and the intracellular fluorescence data were normalized to the plasma membrane fluorescence data (percentage).

## Results

### A Pathogenicity Defect Linked to a Lower Expression of the CHC-Encoding Gene in *Botrytis cinerea*

**FIGURE 1:**
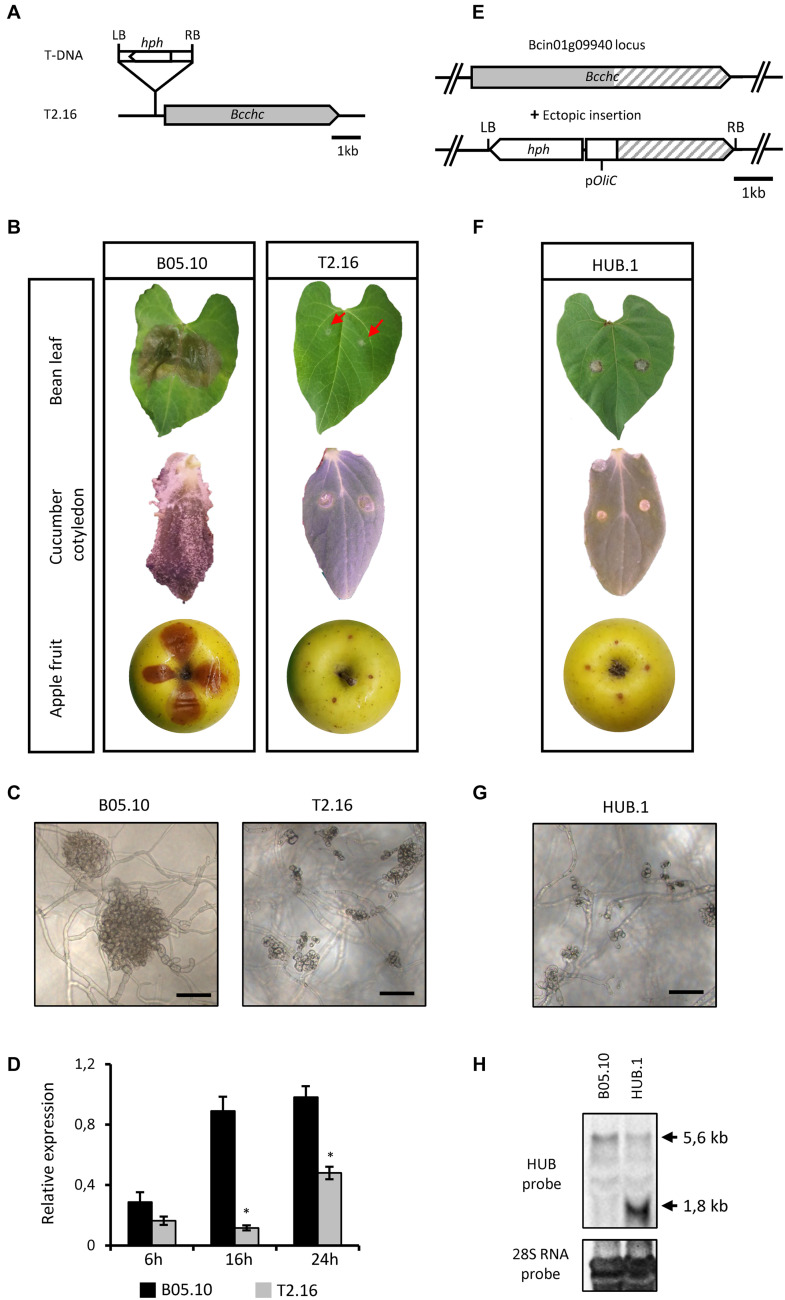
Characterization of the T2.16 and HUB mutants of *B. cinerea*. **(A)** Schematic representation of the mutated locus in the T2.16 strain generated by *A. tumefaciens*-mediated transformation. Insertion of the bacterium T-DNA (*hph* hygromycin B resistance gene flanked by the bacterial left (LB) and right (RB) border regions) occurred 297 bp upstream of open reading frame of the clathrin heavy chain-encoding gene *Bcchc* and was localized by rescue-PCR analysis. **(B,F)** The virulence of the T2.16 and HUB mutants was compared to that of the parental strain B05.10 on primary leaves of French bean, cucumber cotyledons and wounded apple fruits. Pictures were taken 4 days post-inoculation. **(C,G)** Infection cushions were observed by inverted microscopy after 72 h of incubation in liquid PDB 1/4 medium on plastic surfaces (scale bar 50 μm). Photos and data are representative of three independent experiments. **(D)** *Bcchc* expression in the parental (B05.10) and the T2.16 mutant strains during a kinetic of fungal growth on Teflon membranes. Mycelia were harvested at 6 h, 16 h and 24 h after inoculation. *Bcchc* expression is plotted relatively to that of the *Bcpda1* housekeeping gene [the same results were obtained with two other reference genes (see Methods)]. Three independent biological replicates were assessed for each experiment. Standard deviations are indicated. Asterisks indicate a significant difference (Student’s *t*-test, ^∗^*p*-value < 0.05). **(E)** Schematic representation of the DNA construct used to produce the-C-terminal domain of CHC (HUB fragment) in *B. cinerea*. The *A. tumefaciens*-compatible plasmid pBHT2 was used to place the HUB DNA sequence (dashed) under the control of the *A. nidulans* constitutive promoter pOliC. The hygromycin B resistance gene (*hph*) is shown as well as the left (LB) and right (RB) borders of the T-DNA. **(H)** Northern blot analysis. RNA extracted from the parental (B05.10) and HUB.1 strains were separated by gel electrophoresis and hybridized with labeled RNA probes corresponding to the HUB DNA sequence (top) or to the 28S RNA (loading control, bottom). Arrows indicate the hybridizing bands corresponding to the HUB fragment RNA (1.8 kb) and to the *chc* RNA (5.6 kb).

Random mutagenesis of *B. cinerea* through *A. tumefaciens*-mediated transformation has produced a collection of avirulent strains (de Vallée et al., 2019), among which the T2.16 mutant. Southern-blot analysis of this mutant showed a single insertion of the bacterium T-DNA in the fungal genome (Supplementary Figures 1A,C) and a T-DNA rescue approach localized this insertion within the promoter region of the CHC-encoding gene (*Bcchc–*Bcin01g09940), 297 pb upstream of the ATG (Figure 1A). Single-spore isolation coupled to a PCR approach was then used to generate and identify a homokaryotic line (Supplementary Figure 1B). Plant infection assays on different hosts (bean leaves, cucumber cotyledons and apple fruits) confirmed a loss of pathogenicity for the T2.16 mutant while no significant difference in vegetative growth could be observed under *in vitro* conditions (Figure 1B and Supplementary Figure 2A). Besides, microscopic observations showed that the production of infection cushions was altered (smaller and partially developed) in the mutant strain (Figure 1C). Finally, expression of the *Bcchc* gene was investigated by quantitative RT-PCR during a kinetic of spore germination and mycelial growth *in vitro*. This revealed that *Bcchc* was less expressed in the mutant than in the parental strain, up to 10-fold at 16 h post-inoculation (Figure 1D). Altogether, these data indicate that a lower expression of the *Bcchc* gene in *B. cinerea* could impair pathogenicity.

### A Dominant Negative Form of CHC Impairs Virulence in *Botrytis cinerea*

To confirm the link between the pathogenicity of *B. cinerea* and the *Bcchc* gene, the deletion of that gene in the B05.10 strain was attempted. A gene-replacement DNA cassette carrying a hygromycin B resistance gene was introduced into fungal protoplasts and hygromycin-resistant transformants were isolated. Transformants carrying the expected deletion were identified by PCR, but none reached a homokaryotic stage despite several rounds of single-spore isolation (Supplementary Figure 3). As previously observed in many eukaryotic species (Royle, 2006), this result suggests an essential role of *Bcchc* in *B. cinerea*.

Considering the results obtained in mammalian and plant cells (Liu et al., 1995; Adam et al., 2012), an alternative genetic approach was attempted. This approach aimed at altering the cage-forming function of CHC without causing lethal effects on the fungus cells. It is based on the introduction of a DNA sequence coding for a dominant-negative form of the CHC, termed the HUB fragment. This HUB fragment is a truncated version of the CHC that still supports trimerization and the binding of CLC, leading to an open-ended lattice. The potential ability of this fragment to interact with CLC, and the fact that they assemble into non-functional structures, made the HUB fragment a good candidate for a dominant-negative mutant (Liu et al., 1995). Hence, the HUB-encoding DNA sequence was cloned and introduced into the genome of B05.10 under the control of a strong and constitutive promoter (Figure 1E). Independent transformants were isolated and pathogenicity assays revealed a loss of virulence for several of them, and a partial loss of virulence for the others (Figure 1F and Supplementary Figure 2B). Moreover, most of the mutants produced slightly less conidia than the parental strain and microscopic observations revealed that IC development was also altered in the non-pathogenic HUB transformants (Figure 1G). The common avirulent phenotype of the T2.16 mutant and some HUB transformants, produced through different genetic approaches, strongly argued for a link between the fungus pathogenicity and the *Bcchc* gene. One avirulent HUB transformant, hereafter named HUB.1, was selected to verify the expression of the HUB fragment-encoding DNA. In the absence of oligonucleotides that could discriminate the native *Bcchc* locus from the HUB DNA cassette in a qRT-PCR reaction, a northern blot analysis was performed (Figure 1H). Besides the expected similar detection of the *Bcchc* RNA in both the parental and HUB.1 strains, this revealed a strong expression of the HUB fragment-encoding DNA in the HUB.1 strain only. Altogether, these results indicate that the pathogenicity of *B. cinerea* can be altered by a lower expression of the *Bcchc* gene or by the production of a truncated HUB fragment. They also suggest that the correct assembly of clathrin molecules is required to support the fungus virulence.

### Endocytosis Is Not Impaired in the T2.16 and HUB Mutants

**FIGURE 2:**
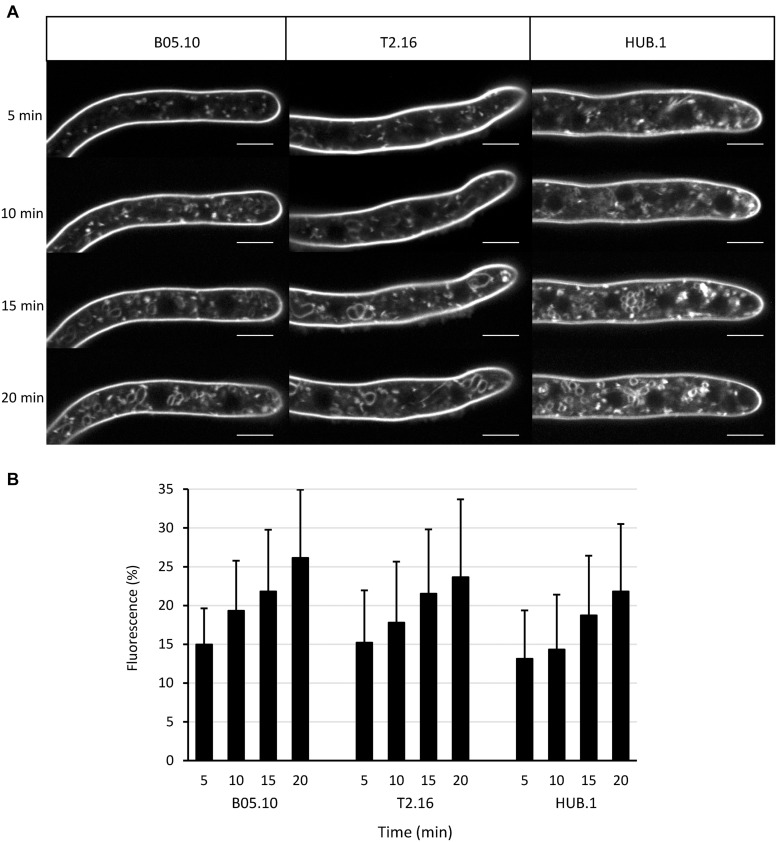
Endocytosis in the clathrin mutants. **(A)** Observation of endocytosis by confocal microscopy. Labeling of the plasma membrane by the fluorescent lipophilic marker FM4-64 and its subsequent cellular internalization were monitored over time in young hyphae (16 h culture) of the parental (B05-10) and mutant (T2.16, HUB.1) strains (scale bar: 5 μm). **(B)** Endocytosis quantification. For 10 different hyphae of each strain, image analysis of the 20 μm apical region led to separate quantifications of the mean total cytoplasmic and mean plasma membrane fluorescence. The plot shows the normalized increase in mean cytoplasmic fluorescence over time (% of plasma membrane fluorescence) in each strain. Standard deviations (10 replicates) are indicated.

Clathrin triskelions are known to play a role in endocytosis by facilitating the invagination of the plasma membrane into CCV (McMahon and Boucrot, 2011). To test whether the reduced expression of *Bcchc* could alter the endocytic process, young mycelia derived from conidia of the parental, T2.16 and HUB.1 strains were incubated with the membrane-selective marker FM4-64 and internalization of the latter was followed by confocal microscopy. After 5 min of incubation, fluorescent dots were detected underneath the labeled plasma-membrane and inside the fungal cells in the three strains (Figure 2A). The size of these intracellular structures (< 1 μm) was compatible with that of putative endosomes, as described in *Neurospora crassa* (Fischer-Parton et al., 2000). At 15 min of incubation, fluorescent circles could be detected inside the hyphae of all strains and their size and/or numbers increased at 20 min (Figure 2A). The shape and size of these intracellular structures were compatible with that of vacuoles. Quantification of the intracellular fluorescence showed an increase of FM4.64 internalization over time that was not significantly different between the parental and mutant strains (Figure 2B). These results indicate that the T2.16 and HUB.1 mutations do not prevent (or reduce) the internalization of FM4-64, a lipophilic dye considered to be internalized by endocytosis in fungi (Read and Kalkman, 2003). Therefore, they suggest that endocytosis is not diminished in the T2.16 and the HUB.1 mutants.

### Clathrin Is Involved in Acidification of Ambient Environment and in Secretion of Hydrolytic Enzymes

**FIGURE 3:**
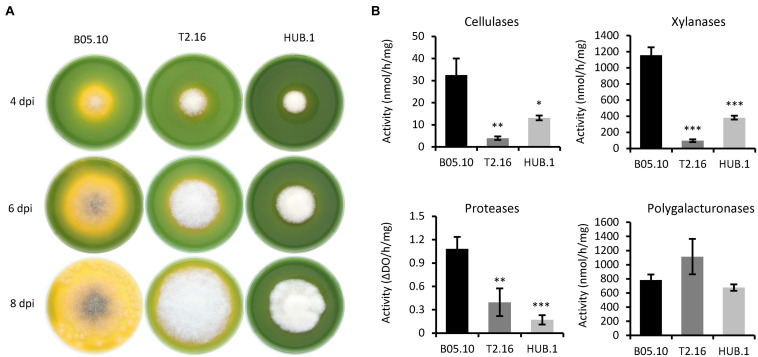
Enzymatic activities and medium acidification in the clathrin mutants. **(A)** Medium acidification. Agar plugs of parental (B05.10) and mutant (T2.16 and HUB.1) mycelia were used to inoculate solid complete medium adjusted to pH 8.0 and supplemented with 0.01% bromothymol blue (yellow at pH < 6.0). Acidification of the medium was monitored at 4, 6, and 8 days post-inoculation. Photos are representative of three independent experiments. **(B)** Enzymatic activities measured in the supernatants of liquid cultures in CCPX medium at 72 h post-inoculation for the parental (B05.10) and mutant (T2.16 and HUB.1) strains. Activities are expressed in nmole or ΔDO per hour per mg of mycelium. Three independent biological replicates were performed. Means with standard deviations are indicated, and asterisks indicate significant difference compared to the B05.10 strain (Student’s *t*-test *p*-value ^∗^ < 0.05; ^∗∗^ < 0.01; ^∗∗∗^ < 0.001).

*Botrytis cinerea* is known to modulate the ambient pH during the infectious process by secreting organic acids (Billon-Grand et al., 2012). This modulation contributes to the intoxication of plant cells and to the efficient action of the fungal lytic enzymes. The capacity to acidify the environment can be visualized *in vitro* on rich medium agar plates containing bromothymol blue as a pH indicator (Schumacher et al., 2014). As shown in Figure 3A, growth of the T2.16 mutant led to a reduced green-to-yellow change in the corresponding agar plates compared to the control plates. An even stronger phenotype was observed in the case of the HUB.1 mutant (Figure 3A) and these results suggest that the two mutations affecting clathrin in the T2.16 and HUB.1 mutants alter the secretion of organic acids.

The necrotrophic strategy of *B. cinerea* is partly based on the secretion of a large set of hydrolytic enzymes that contribute to the killing and degradation of plant cells. Since clathrin can be involved in the formation of secretory vesicles from the Golgi apparatus (Robinson, 2015), the secretory capacity of the T2.16 mutant was therefore investigated. The mutant being non-pathogenic, secreted fungal molecules could not be retrieved from infected plant tissues and *in vitro* cultures were therefore used. First, the mutant and parental strains were grown in liquid medium for 3 days and the culture media were used in enzymatic assays to test for the presence of cellulases, xylanases (hemicellulases), proteases and polygalacturonases. To promote enzyme secretion, a synthetic liquid medium containing polysaccharides and casein as nutrients was used (CCPX medium; de Vallée et al., 2019). Under these conditions, less enzymatic activities were measured in the culture media of the T2.16 mutant compared to that of the parental strain: 8.5, 12.5, and 6.4-fold reduction of cellulase, xylanase and protease activity, respectively (Figure 3B). In contrast, the culture media of the T2.16 mutant showed no significant different polygalacturonase activity (1.4×) in comparison to that of the parental strain. These results were indicative of a deficiency in the secretion of some hydrolytic enzymes in the T2.16 mutant and similar observations in the HUB.1 mutant (Figure 3B) strengthened this hypothesis.

### Secretion of ROS Is Impaired in the Clathrin Mutants

**FIGURE 4:**
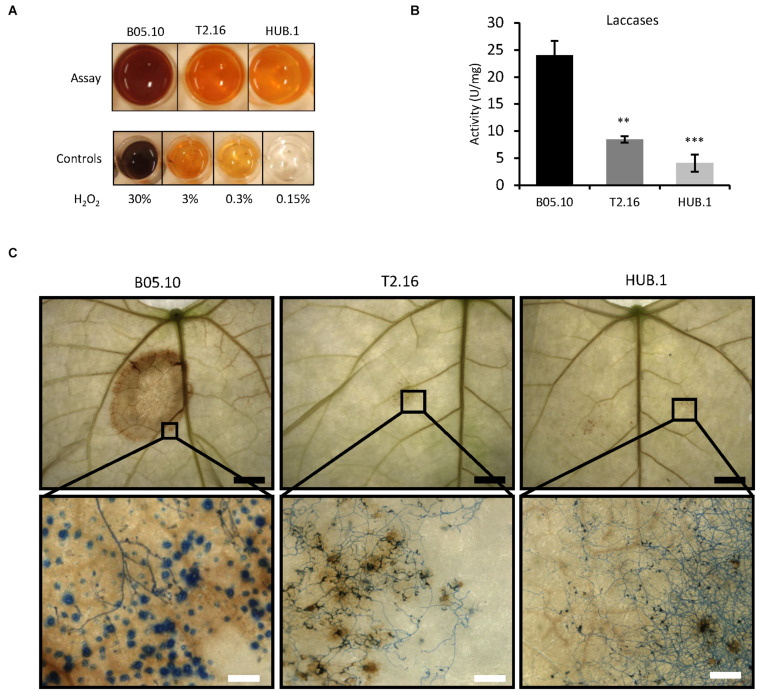
Secretion of ROS in the clathrin mutants. **(A)** Production of ROS *in vitro*. Equal quantities of mycelia from the parental (B05.10) and mutant (T2.16 and HUB.1) strains were incubated 2 h in the presence of soluble colorless DAB. DAB oxidation into a brown precipitate was monitored and compared to controls using defined quantities of H_2_O_2_. **(B)** Laccase activities measured in the supernatant of liquid cultures in CCPX medium at 72 h for the parental (B05.10) and the mutant (T2.16 and HUB.1) strains. Activities are expressed in arbitrary units (U) per mg of mycelium, three independent biological replicates were performed. Standard deviations are indicated, and asterisks indicate significant difference compared to the B05.10 strain (Student’s *t*-test *p*-value ^∗∗^ < 0.01; ^∗∗∗^ < 0.001). **(C)** ROS detection *in planta*. Primary bean leaves were inoculated with conidia of the parental (B05.10) and mutant (T2.16 & HUB.1) strains and stained with DAB and cotton blue in lactic acid (beta-glucan targeting dye) at 72 h post-infection. DAB oxidation into brown precipitates and hyphal coloration were monitored using a macroscope at different magnifications (black scale bar 5 mm; white scale bar 200 μm). Blue dots in the parental sample could represent callose depositions by the plant (also made of beta-glucans).

During the first steps of the infectious process of *B. cinerea*, reactive oxygen species (ROS) play a dual role. They are generated by the pathogen to intoxicate the host-plant tissues, but they are also produced by the plant as part of its defense reaction (Siegmund and Viefhues, 2016). On the fungal side, ROS are produced and accumulate in the extracellular medium, partly through the action of several secreted oxidases including laccases (Siegmund and Viefhues, 2016). To evaluate the impact of the *Bcchc* reduced expression on fungal ROS production, the parental, T2.16 and HUB.1 strains were grown on solid complete medium and the oxidative activity produced by the mycelia was measured. As shown in Figure 4A, oxidation of diaminobenzidine (DAB) was affected in the T2.16 and HUB.1 mutants, indicating reduced H_2_O_2_ production compared to the parental strain. As a complement to these data, laccase activities were quantified in the same culture media used to measure the secreted enzymatic activities presented in Figure 4, and this revealed that the T2.16 and HUB.1 mutants secreted less laccases than the parental strain (a three and six times fold reduction, respectively) (Figure 4B).

ROS detection was then performed *in planta* to evaluate the plant reaction to the mutant strains. Primary bean leaves inoculated with conidia of the parental or mutant strains were collected at 72 h of infection and stained with DAB (Figure 4C, top). In leaves inoculated with conidia of the parental strain, the DAB oxidation by the ROS present in the infected tissues yielded a distinct and large brown zone. In comparison, the leaves inoculated with conidia of the T2.16 mutant developed a barely visible brown coloration. Subsequent staining of the fungal cells with Cotton blue and observations at higher magnification revealed the presence of ROS throughout the plant tissues colonized by the parental strain (Figure 4C, bottom), up to the edge of the infected area. As for the T2.16 mutant, these observations showed that the conidia had germinated onto the leaves surface and had produced hyphae, underneath which only few brown spots of small size were visible. These results demonstrate that the lack of virulence of the T2.16 mutant is not due to a conidial germination default or to an impaired hyphal growth at the plant surface. The very small amounts of ROS produced at the interface between the plant and the T2.16 mutant were consistent with the weak ROS production measured *in vitro* (see above) and moreover indicates that the plant does not produce much ROS in response to the presence of mutant hyphae. Finally, the HUB.1 mutant behaved similarly to the T2.16 mutant with respect to laccase activity and ROS production *in vitro* and *in planta* (Figure 4). Altogether with the results presented in Figure 3, these data led to the hypothesis of clathrin playing an important role in the secretion of numerous factors required for virulence in *B. cinerea*.

### The Exo-Proteome of *Botrytis cinerea* Is Modified in the T2.16 Mutant

A deeper exploration of the impact of the *Bcchc* reduced expression on the fungal secretion was achieved through a comparative shotgun proteomic analysis. The parental and T2.16 mutant strains were grown in liquid CCPX medium and the proteins extracted from the culture supernatants were analyzed by mass spectrometry. In total, 745 proteins were identified, but the analysis was restricted to 203 proteins detected in the three biological replicates of each strain, with a minimum of two unique peptides (Supplementary Table 2). Based on signal peptide (SP) prediction (Almagro Armenteros et al., 2019), these 203 proteins were divided into 178 SP-containing proteins (87.6%) and 25 proteins predicted not to contain a SP (Supplementary Table 2). Of the 178 SP-containing, 59 (33.1%) showed a similar accumulation in the mutant and parental exo-proteomes while 68 (38.2%) and 51 (28.6%) were, respectively, down- and up-accumulated in the mutant exo-proteome (Supplementary Table 2). At first, this indicates that the lower expression of the *Bcchc* gene has a significant impact on the secretion of SP-containing proteins in *B. cinerea*. Considering the 25 proteins without SP, 11 showed a similar accumulation in the mutant and parental exo-proteomes, 11 were down-accumulated in the mutant exo-proteome and three were up-accumulated (Supplementary Table 2).

**FIGURE 5:**
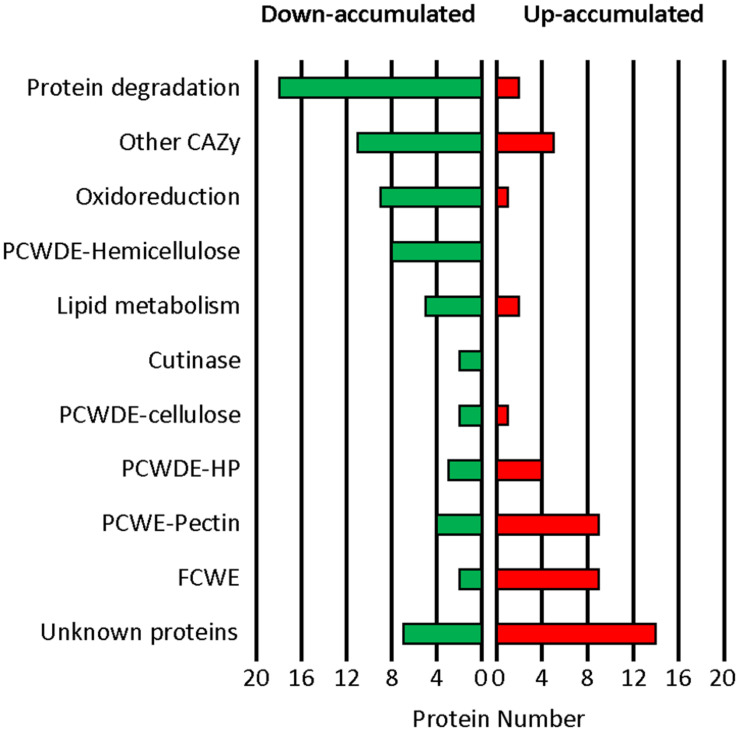
Impact of the T2.16 mutation on the exo-proteome of *B. cinerea.* Functional categories classification of the secreted proteins that were down- or up-accumulated in comparison to the parental control (B05.10) (fold change > 2), (see Supplementary Table 2 for details). The parental and the T2.16 mutant strains were grown for 3 days in liquid CCPX medium (three independent biological experiments) and a comparative shotgun proteomic analysis was performed on the proteins collected from the cultures supernatants. CAZy, carbohydrate active enzymes; PCWDE, plant cell wall degrading enzymes; HP, Hemicellulose-Pectin; FCWE, fungal cell wall enzymes.

Following functional classification (Supplementary Table 2), comparison of the datasets revealed less proteases, less CAZy targeting plant cell wall components and less oxidoreductases in the mutant exo-proteome than in the parental counterpart (Figure 5). These down-accumulations were consistent with the previously measured enzymatic activities and ROS productions (Figures 3, 4). Protein degradation represented the function the most affected by the mutation as 18 proteases were down-accumulated in the exo-proteome of the T2.16 mutant. These proteases include aminopeptidases, subtilisins, carboxypeptidases and the deuterolysin Bcin12p06300 which accumulated 250-fold less in the mutant than in the parental exo-proteome. Hemicellulose-degradation also emerged as a function strongly affected by the mutation as eight xylanases were down-accumulated in the exo-proteome of the T2.16 mutant. These xylanases include BcXyn10A, BcXyn11A, and BcXyn10B that, respectively, accumulated 24, 6, and threefold less in the mutant than in the parental exo-proteome. Furthermore, three plant cell wall degrading enzymes that could also target hemicellulose (PCWDE-HP) and 11 “other CAZy” were less abundant in the mutant exo-proteome. Oxidoreduction was the third function that emerged to be negatively affected by the mutation as nine predicted oxidoreductases were less accumulated in the mutant than in the parental exo-proteome. These proteins include the two glyoxal oxidases BcGO1 and BcGO2, the dioxygenase BcIDL1 and the laccase BCLCC9. At the other end of the spectrum, nine fungal cell wall enzymes (FCWE), nine pectinases and four PCWDE-HP appeared more abundant in the mutant exo-proteome than in the parental counterpart. The pectinases include the endopolygalacturonases BcPG2, BcPG4 and BcPG6, the exopolygalacturonase BcPGX1 and the pectin methyl esterases BcPME2. Noticeably, this up-accumulation contrasts with the moderate increase in polygalacturonase activity that was measured in the mutant culture supernatants (Figure 3). Altogether, and in addition to the down-accumulation of two cutinases in the mutant exo-proteome, these data reveal that a lower expression of the *Bcchc* gene impacts on the fungus capacity at secreting multiple enzymes known to play a role in the infectious process. Furthermore, the T2.16 mutation led to down-accumulation of four known virulence factors, three of them being cell death inducing proteins (CDIPs): the glycoprotein BcEb1 (Frías et al., 2016), the glucoamylase BcGs1 (Zhang et al., 2015) and the xylanase BcXyn11A (Brito et al., 2006) (Supplementary Table 2). Finally, we noticed that seven proteins were totally missing from the mutant exo-proteome while present in the parental counterpart (Supplementary Table 2). In particular, the mutant exo-proteome lacked 1 oxido-reductase, 1 putative lipase, 1 CAZy (GH92) and 1 chitinase (GH18). Conversely, five proteins over-accumulated in the mutant exo-proteome while being absent in the exo-proteome of the parental strain: 1 fungal cell wall protein, 1 CAZy (GH135) and three proteins of unknown function. This indicates that the reduced expression of *Bcchc* can dramatically alter the secretion of some proteins.

## Discussion

### Clathrin and Pathogenicity

To date, little is known about the role of clathrin in filamentous fungi despite the importance of vesicular trafficking in fungal growth (Shoji et al., 2014). In addition, there is no report describing the importance of clathrin in the infectious process of plant pathogenic fungal species. The isolation of a non-pathogenic mutant of *B. cinerea* altered in the expression of the CHC-encoding gene led us to investigate the role of clathrin in the virulence of this necrotrophic fungus.

In eukaryotes, the heavy (CHC) and light (CLC) chains of clathrin can assemble into triskelions that, in turn, can form cages at sites of exocytic and/or endocytic vesicle production. Clathrin is also involved in cellular processes important for cell division, such as mitotic and meiotic spindle organization and stabilization (Liu and Zheng, 2009; Hölzenspies et al., 2010), Golgi integrity and segregation (Radulescu and Shields, 2012), segregation of mitochondria (Pieperhoff et al., 2013) or cytokinesis (Niswonger and O’Halloran, 1997; Van Damme et al., 2011). This could explain why clathrin has been found essential in numerous eukaryotes, including some in which endocytosis could be clathrin-independent (Royle, 2006). Here we report on our attempt to create a strain of *B. cinerea* deleted of the CHC-encoding gene *Bcchc*. Despite correct replacement of that gene by a hygromycin B resistance cassette, no homokaryotic line could be obtained, and this suggests that clathrin is also essential to cell division and/or growth in *B. cinerea*.

We identified a T-DNA insertion in the promoter region of the *Bcchc* gene (T2.16 mutation) that led to a reduced expression of that gene (up to 10-fold). Interestingly, the mutation appeared compatible with fungal growth *in vitro* while abolishing virulence on different host plants. This indicates that clathrin carries a function essential for virulence in *B. cinerea* besides its essential function in cell division/growth. It further indicates that the amount of CHC present in the mutant cells, likely reduced because of the mutation, is sufficient to support growth and division *in vitro*. Incidentally, we did not observe a negative impact of the T2.16 mutation on endocytosis, a process that contributes to fungal growth (Riquelme et al., 2018) and that was shown to involve clathrin in the closely related fungus *S. sclerotiorum* (Wytinck et al., 2020). The amount of CHC present in the mutant cells may therefore be sufficient to support endocytosis. Alternatively, a clathrin-independent endocytic pathway might exist in *B. cinerea*, as proposed in *A. nidulans* (Schultzhaus et al., 2017).

To consolidate the results obtained with the mutant exhibiting a reduced expression of the *Bcchc* gene, we created new strains of *B. cinerea* that expressed a HUB fragment-encoding DNA. This fragment (C-terminal third of CHC) competes with intact CHC to bind to CLC, and it therefore acts as a dominant negative inhibitor of clathrin cage formation (Liu et al., 1995). This approach has been validated in mammalian, plant and protist cells and is acknowledged as a standard approach to study clathrin function (Liu et al., 1998; Bennett et al., 2001; Kitakura et al., 2011; Pieperhoff et al., 2013). *B. cinerea* HUB mutants were obtained, and they were impaired in virulence. Together with the T2.16 phenotype, this constitutes a strong evidence that clathrin plays a critical role in the pathogenicity of this fungus. Virulence was either severely reduced or abolished in the HUB mutants and the difference could be due to various expression levels of the *trans-*gene following ectopic integrations of the HUB DNA cassette in the fungal genome. We also observed that the radial growth of the HUB mutants was slightly reduced *in vitro*. We suppose that the level of HUB fragments production that is required to abolish virulence disturbs cellular processes related to fungal growth. This would be consistent with the essential role of clathrin in cell growth and the reported lethal effect of a strong expression of the HUB fragment-encoding DNA (Pieperhoff et al., 2013). Under the conditions we used to screen the HUB mutants, such lethal expression level was counter-selected and could therefore not be observed.

Finally, microscopic studies revealed that both the T2.16 and HUB.1 mutants were altered in the production of infection cushions (IC), multicellular appressoria dedicated to the massive secretion of effectors during plant infection (Mentges et al., 2020; Choquer et al., 2021). Based on the important role IC play in the virulence of *B. cinerea* (de Vallée et al., 2019), this default could explain the loss of pathogenicity in these mutants. Besides, this result suggests that clathrin participates in the differentiation process of IC, possibly through the formation and/or trafficking of vesicles involved in hyphal growth, or in the transport of IC-specific actors. This would compare to the parasite *Toxoplasma gondii* whose development inside host cells is aberrant in HUB mutants (Pieperhoff et al., 2013). In this pathogen, clathrin is essential to vesicle formation at the *trans-*Golgi and is involved in the biogenesis of the apical secretory organelle.

### Clathrin and the Secretion of Plant Cell Wall Degrading Enzymes

*Botrytis cinerea* is considered a typical necrotrophic pathogen that feeds on killed plant cells through the secretion of hydrolytic enzymes. In the culture medium of both the T2.16 and HUB.1 mutants, xylanase, protease and cellulase activities were reduced when compared to the parental control, and this was indicative of a decreased secretion of lytic enzymes in these strains. Through quantitative proteomics analysis of the T2.16 mutant exo-proteome, it was possible to show that the secretion of 68 proteins with a signal peptide was negatively affected by the lower expression of the *Bcchc* gene. In particular, the secretion of 18 proteases and 32 carbohydrate-active enzymes (CAZy) including cutinases, xylanases, cellulases and pectinases was modified. This suggests that clathrin plays a significant role in the secretion of PCWDE.

### Clathrin and the Secretion of Necrosis Inducers

The initial stage of plant infection by *B. cinerea* is characterized by local necrotic lesions. These lesions correspond to patches of dead plant cells and result from the secretion of plant cell death-inducing factors by the pathogen. Secreted organic acids (i.e., oxalic acid) are such factors (Verhoeff et al., 1988; Kim et al., 2008; Rascle et al., 2018) and both the T2.16 and HUB.1 mutants were impaired in their capacity at acidifying their ambient environment. Besides metabolites, *B. cinerea* also produces proteins that can induce a hypersensitive-like response (Li et al., 2020). Analysis of the fungal exo-proteome revealed that the secretion of three known apoplastic cell death-inducing proteins (CDIPs) was reduced in the T2.16 mutant strain: the small glycoprotein BcIeb1 (Frías et al., 2016), the 1,4 alpha glucosidase BcGs1 (Zhang et al., 2015) and the xylanase BcXyn11A (Brito et al., 2006). Altogether, this indicates that the secretion of some necrosis inducers is clathrin-dependent and its reduction in the T2.16 mutant could contribute to its avirulent phenotype. Interestingly, the production of key virulence factors is also affected in clathrin mutants of the encapsulated yeast *Cryptococcus neoformans* (Bairwa et al., 2019).

### Clathrin and the Plant-Fungus ROS Interface

The plant’s oxidative burst is a primary defense system against aggressors like fungi, but the production of harmful reactive oxygen species (ROS) is also broadly used by necrotrophic fungi in the course of infection. To produce and to cope with ROS, *B. cinerea* is equipped with multiple oxidoreductases (for review, see Siegmund and Viefhues, 2016). On one hand, several oxidases and laccases have been identified as ROS producers at the plant interface. On the other hand, *B. cinerea* relies on glutathione reductases, thioredoxin reductases and extra-cellular ROS scavenging enzymes such as catalases, superoxide dismutases, peroxiredoxins and peroxidases to protect itself against ROS. When the T2.16 and HUB.1 mutants were grown *in vitro*, less ROS and less laccase activity were recorded in their supernatants as compared to the parental counterpart. In addition, when the mutants were confronted to bean leaves, ROS could barely be detected in the plant tissues whereas a strong accumulation of ROS was revealed in leaves infected by the parental strain. In support to this, our comparative proteomics analysis showed that two predicted oxidases were totally missing from the T2.16 exo-proteome and seven predicted secreted oxidases (including 1 laccase and 2 glyoxal oxidases) were down-accumulated when compared to the parental exo-proteome. Altogether, this suggests that clathrin also plays a role in the delivery of ROS producing enzymes. Interestingly, the quasi-absence of ROS production by leaves confronted to the T2.16 and HUB.1 mutants suggests that their avirulent phenotype does not result from an arrest of the fungal growth by the plant oxidative burst. It rather suggests that the clathrin mutants are not detected by the plant, possibly because of the default in IC production and plant penetration and/or the default in oxalic acid, CDIPs, PCWDE and ROS secretion.

### Clathrin-Independent Secretion

Our comparative proteomics analysis led to the listing of 70 proteins whose secretion was unaffected by the T2.16 mutation. Furthermore, it revealed 54 proteins that were up-accumulated in the mutant exo-proteome, including notably nine pectinases and nine fungal cell wall enzymes. Whether the secretion of these proteins could rely on clathrin-independent secretory vesicles is an open question and further investigations are needed to better understand the vesicular traffic leading to secretion in *B. cinerea*.

## Conclusion

This study shows that clathrin acts as an essential player in the infectious process of the phytopathogenic fungus *B. cinerea*. It reveals that this protein takes part in the secretion of cell death inducing proteins (CDIPs), proteins associated with ROS production and plant cell wall degrading enzymes. It further shows the importance of clathrin in the development of infection cushions, structures dedicated to the penetration and early destruction of the host tissues by the pathogen.

## Data Availability Statement

The datasets presented in this study can be found in online repositories. The names of the repository/repositories and accession number(s) can be found below: http://www.proteomexchange.org/, PXD023460.

## Author Contributions

NP, ES, and M-PL initiated the research project. ES and NP defined the research question and designed the experiments. ES, CR, and AV conducted the experimental studies. ES, CD, and JD conducted the proteomic experiments. ES, CB, and NP analyzed the proteomic data. ES, NP, CB, and MC wrote the manuscript. All authors commented on and approved the final draft of the manuscript.

## Conflict of Interest

M-PL was employed by the company Bayer SAS. The remaining authors declare that the research was conducted in the absence of any commercial or financial relationships that could be construed as a potential conflict of interest.

## Supplementary Material

Supplementary Figure 1 Genotypic characterization of the T2.16 mutant: **(A)** Schematic representation of the T2.16 insertion mutant locus**. (B)** Validation of the homokaryotic line by PCR amplification using primers P1 and P2 (Supplementary Table 1). **(C)** Southern blot analysis. Genomic DNA was digested with *Nco*I and probed with a digoxigenin-labeled DNA fragment corresponding to the *hph* gene (black box in A). T2.16 strain shows single T-DNA insertion. Black arrows indicate the sizes of the T2.16 hybridized fragments (2.56 and 14.9 Kb). (^∗^) ATMT-transformants strains not retained in this study. LB, left border; RB, right border.

Supplementary Figure 2 Characterization of HUB mutants. **(A)** Radial growth of the parental and mutant (T2.16 and 4 HUB) strains after 4 days on sporulation medium. Means of three independent experiments are shown with standard deviations and statistic significant differences compared to the parental strain (Student *t*-test; ^∗∗∗^*p*-value < 0.001). **(B)** Pathogenicity assays. The virulence of four HUB mutants was compared to that of the parental (B05.10) and T2.16 mutant strains on primary leaves of French bean and cucumber cotyledons. Agar explants of young mycelia were used as inocula and pictures were taken 4 days post inoculation. Pictures are representative of three independent experiments.

Supplementary Figure 3 Identification of *B. cinerea Bcchc* null mutants. **(A)** Schematic representation of the *Bcchc* gene replacement by the hygromycin B resistance cassette (*hph*) flanked by 1,03 kb of 5′ and 0,84 kb of 3′ sequences from the *Bcchc* locus. Primers (black arrows; Supplementary Table 1) used for PCR analysis are indicated. **(B)** PCR amplification of the expected 5′ (top) and 3′ (bottom) regions of the deleted *Bcchc* locus in nine independent transformants (WT, parental control). The primer pairs used and the sizes of the expected bands in the transformants carrying the gene deletion are indicated **(C)** PCR amplification of the *Bcchc* gene revealing its presence in all transformants despite several rounds of single-spore isolation. The primers pairs and the expected band size in the control (WT) are indicated.

## Abbreviations

CHC: Clathrin Heavy Chain
ATMT: Agrobacterium tumefaciens-mediated transformation
PM: Plasma Membrane
TGN: Trans Golgi Network
CCV: Clathrin Coat Vesicles
CLC: Clathrin Light Chain
CME: Clathrin Mediated Endocytosis
ROS: Reactive Oxygen Species
SP: Signal Peptide
CAZy: Carbohydrate Active Enzymes
PCWDE: Plant Cell Wall Degrading Enzymes
FCWE: Fungal Cell Wall Enzymes
CDIPs: Cell Death-Inducing Proteins.
