# Supplementary figures and images for: Clathrin Is Important for Virulence Factors Delivery in the Necrotrophic Fungus Botrytis cinerea
Source: Front Plant Sci. 2021 Jun 16;12:668937. doi: 10.3389/fpls.2021.668937 (PMC8244658; doi:10.3389/fpls.2021.668937)

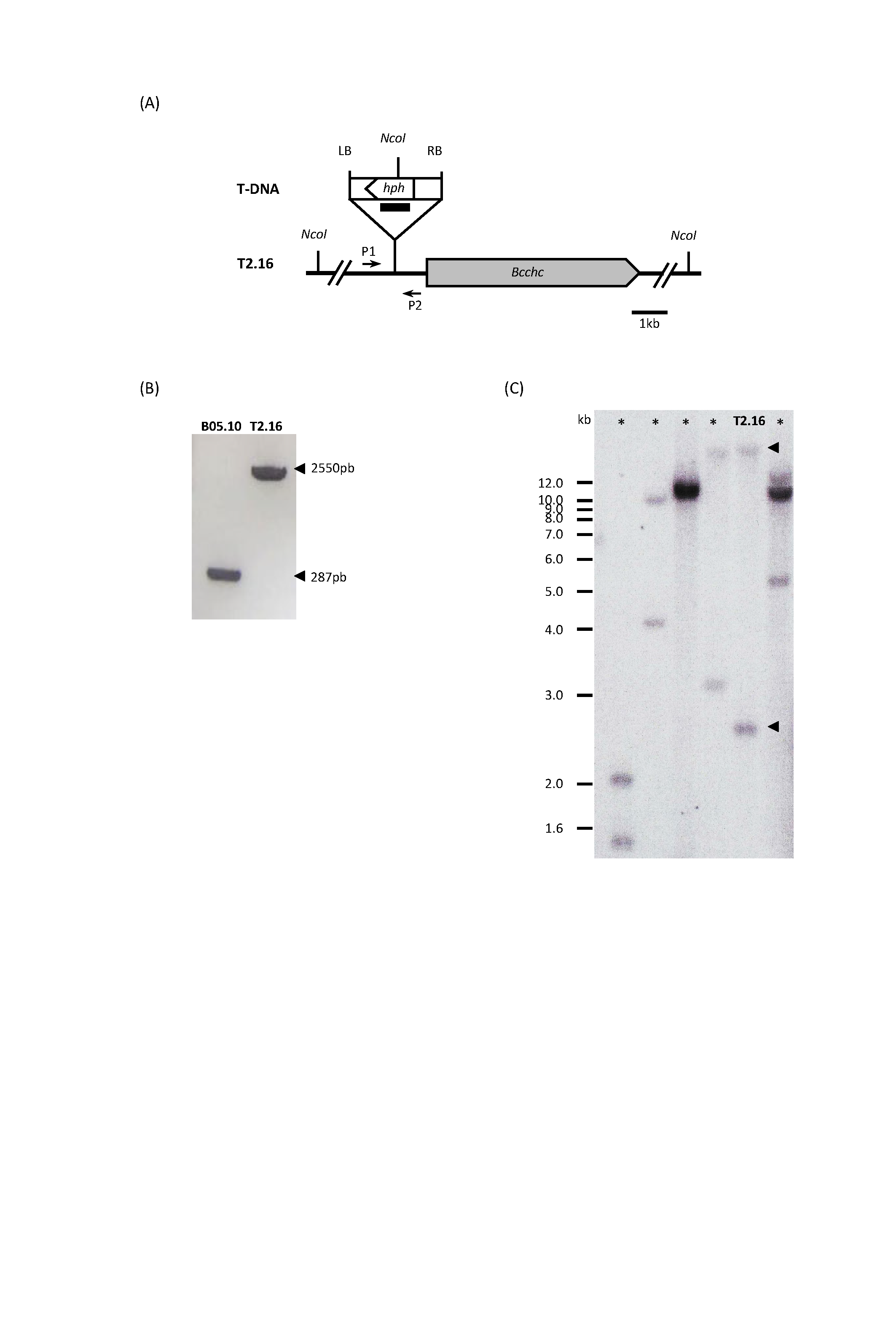

Supplement: Supplementary Figure 1 — Genotypic characterization of the T2.16 mutant: (A) Schematic representation of the T2.16 insertion mutant locus. (B) Validation of the homokaryotic line by PCR amplification using primers P1 and P2 (Supplementary Table 1). (C) Southern blot analysis. Genomic DNA was digested with NcoI and probed with a digoxigenin-labeled DNA fragment corresponding to the hph gene (black box in A). T2.16 strain shows single T-DNA insertion. Black arrows indicate the sizes of the T2.16 hybridized fragments (2.56 and 14.9 Kb). (∗) ATMT-transformants strains not retained in this study. LB, left border; RB, right border. [file Image_1.TIF]

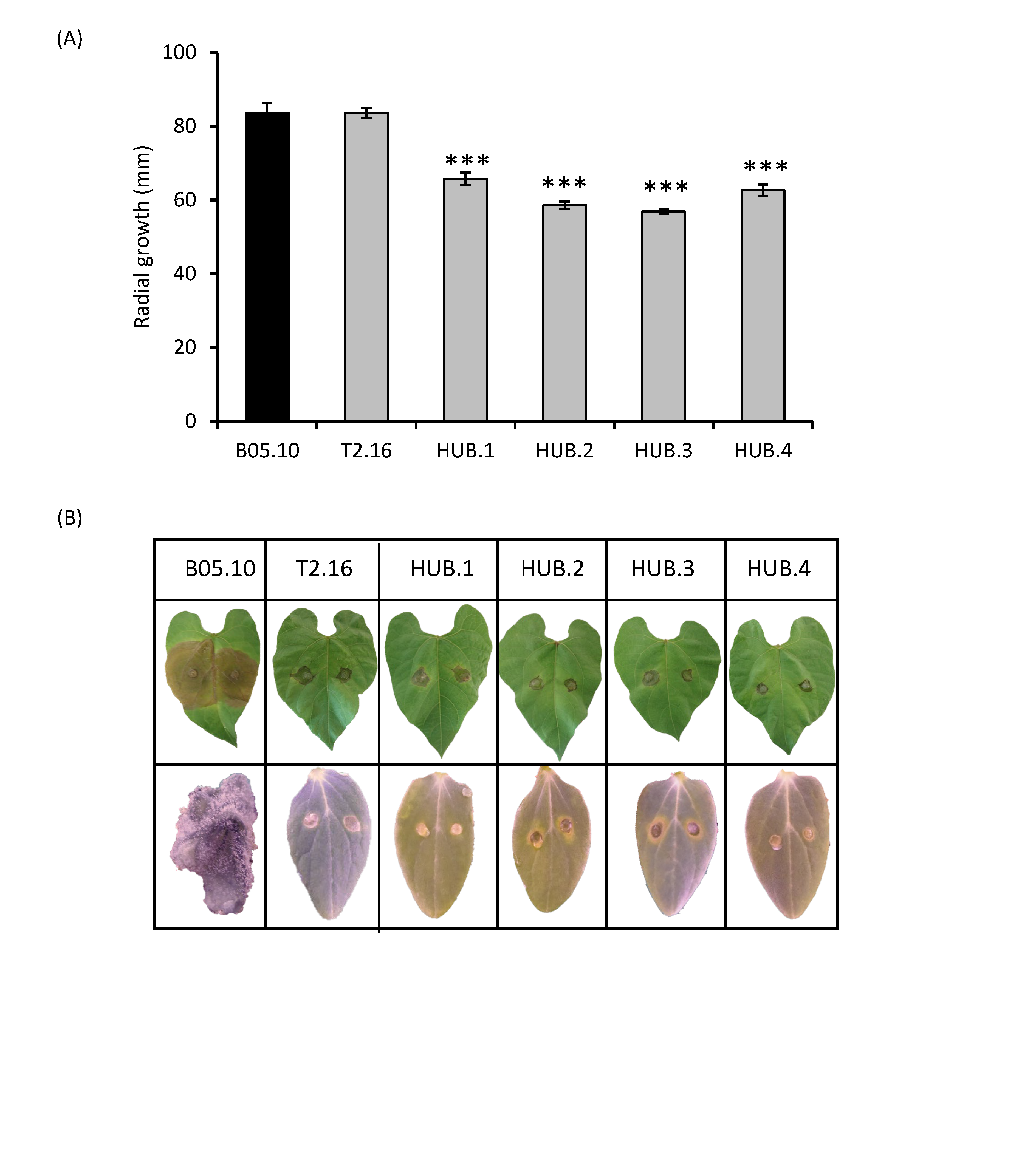

Supplement: Supplementary Figure 2 — Characterization of HUB mutants. (A) Radial growth of the parental and mutant (T2.16 and 4 HUB) strains after 4 days on sporulation medium. Means of three independent experiments are shown with standard deviations and statistic significant differences compared to the parental strain (Student t-test; ∗∗∗p-value < 0.001). (B) Pathogenicity assays. The virulence of four HUB mutants was compared to that of the parental (B05.10) and T2.16 mutant strains on primary leaves of French bean and cucumber cotyledons. Agar explants of young mycelia were used as inocula and pictures were taken 4 days post inoculation. Pictures are representative of three independent experiments. [file Image_2.TIF]

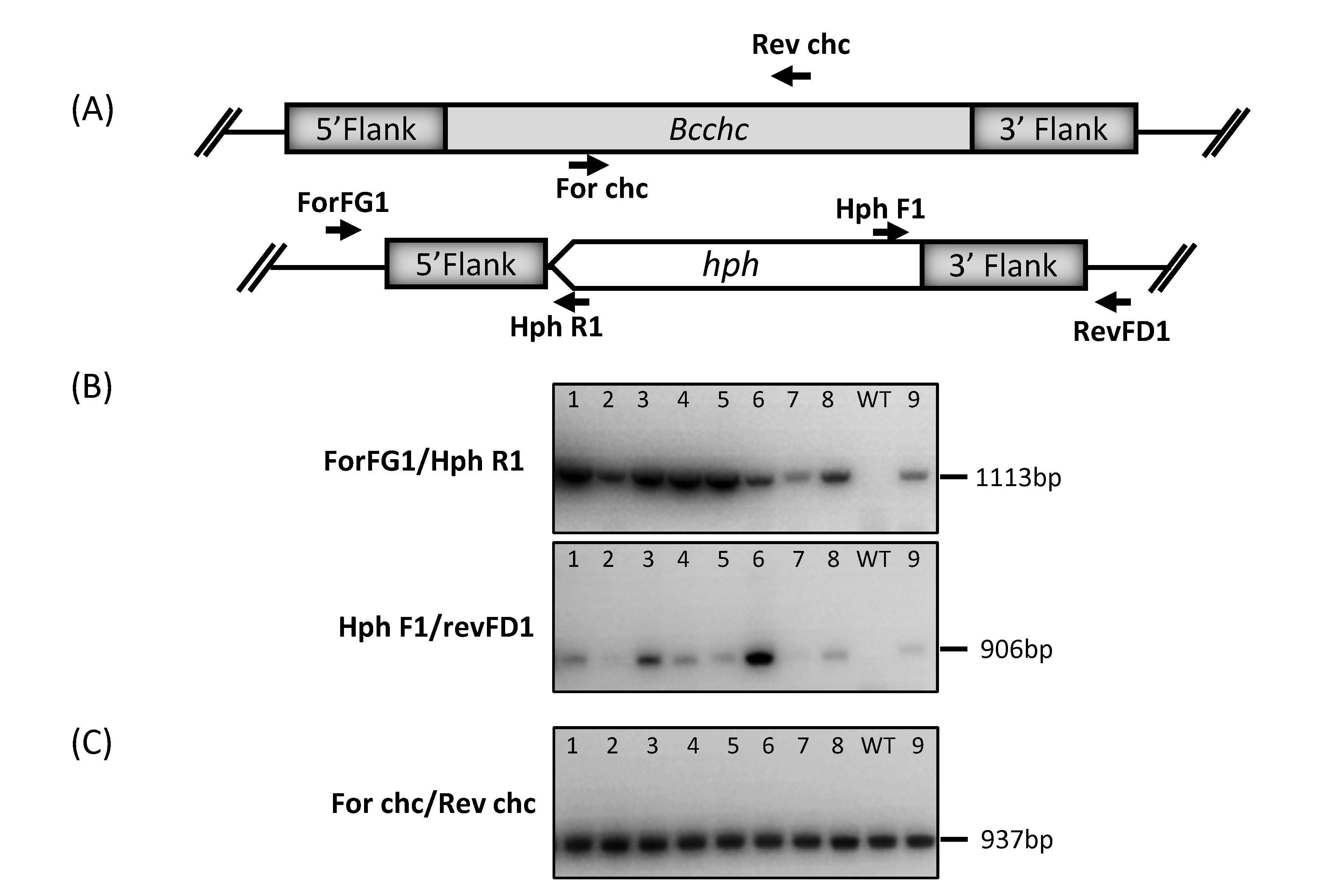

Supplement: Supplementary Figure 3 — Identification of B. cinerea Bcchc null mutants. (A) Schematic representation of the Bcchc gene replacement by the hygromycin B resistance cassette (hph) flanked by 1,03 kb of 5′ and 0,84 kb of 3′ sequences from the Bcchc locus. Primers (black arrows; Supplementary Table 1) used for PCR analysis are indicated. (B) PCR amplification of the expected 5′ (top) and 3′ (bottom) regions of the deleted Bcchc locus in nine independent transformants (WT, parental control). The primer pairs used and the sizes of the expected bands in the transformants carrying the gene deletion are indicated (C) PCR amplification of the Bcchc gene revealing its presence in all transformants despite several rounds of single-spore isolation. The primers pairs and the expected band size in the control (WT) are indicated. [file Image_3.TIF]
