# Supplementary material for: Clathrin Is Important for Virulence Factors Delivery in the Necrotrophic Fungus Botrytis cinerea
Source: Front Plant Sci. 2021 Jun 16;12:668937. doi: 10.3389/fpls.2021.668937 (PMC8244658; doi:10.3389/fpls.2021.668937)
Supplement: Supplementary file 4 [file Table_1.DOCX]

| Table S1. Primers used in this study | |
| --- | --- |
|  | |
| **Name** | **Sequence 5'→3'** |
| **Constructs** | |
| P1 | ATTGATAGCTTCATCTTCGC |
| P2 | TGAAAGTACAAGTGTTGTGGG |
| P6 | CATCACATCACAATCGATCCCCATGAAGATTAACGACCACATCAACGCT |
| P8 | ACTGACTCCTGCAGGATTGACATACCATACCGGATCTTCC |
| ForFG1 | GCTTATGACGACAGGCTCCG |
| RevFD1 | ACATCACATCACATCCATTCC |
| Hph R1 | CCCGGTCGGCATCTACTCTA |
| Hph F1 | CCACTAGCTCCAGCCAAGCC |
| For chc | TTACTTTGGTCGCTTGTCCC |
| Rev chc | CGCTCTAAACAAAAGGACGG |
|  |  |
| **Northern blot** | |
| 28S-R | TAATACGACTCACTATAGGGAGAAACACCACTTTCTGGCCATC |
| 28S-F | TAATACGACTCACTATAGGGAGAAACAGCAGTTGGACATGGGT |
| HUB-R | TAATACGACTCACTATAGGGAGACCGCAATAAGCTCATCGAAA |
| HUB-F | TAATACGACTCACTATAGGGAGAAGATTAACGACCACATCAACGC |
|  |  |
| **qPCR** | |
| pda1-F | CGCTGTTAAGGCTGCTGTCA |
| pda1-R | CGAGGACTAATGGACCGTTACC |
| P3 | CCCTCTCATCAAACCCTTCCT |
| P4 | TCATGGATGGCACTGTTGACA |
|  |  |
| **Southern blot** | |
| hph15 | CAAGCTGCATCATCGAAATTGC |
| hph21 | ATCGAAAAGTCCGACAGCGTC |
